# Inhibition of Aerobic Glycolysis Attenuates Disease Progression in Polycystic Kidney Disease

**DOI:** 10.1371/journal.pone.0146654

**Published:** 2016-01-11

**Authors:** Meliana Riwanto, Sarika Kapoor, Daniel Rodriguez, Ilka Edenhofer, Stephan Segerer, Rudolf P. Wüthrich

**Affiliations:** 1 Nephrology, Institute of Physiology, University of Zurich, Zurich, Switzerland; 2 Division of Nephrology, University Hospital Zurich, Zurich, Switzerland; University of Geneva, SWITZERLAND

## Abstract

Dysregulated signaling cascades alter energy metabolism and promote cell proliferation and cyst expansion in polycystic kidney disease (PKD). Here we tested whether metabolic reprogramming towards aerobic glycolysis (“Warburg effect”) plays a pathogenic role in male heterozygous Han:SPRD rats (Cy/+), a chronic progressive model of PKD. Using microarray analysis and qPCR, we found an upregulation of genes involved in glycolysis (*Hk1*, *Hk2*, *Ldha*) and a downregulation of genes involved in gluconeogenesis (*G6pc*, *Lbp1*) in cystic kidneys of Cy/+ rats compared with wild-type (+/+) rats. We then tested the effect of inhibiting glycolysis with 2-deoxyglucose (2DG) on renal functional loss and cyst progression in 5-week-old male Cy/+ rats. Treatment with 2DG (500 mg/kg/day) for 5 weeks resulted in significantly lower kidney weights (-27%) and 2-kidney/total-body-weight ratios (-20%) and decreased renal cyst index (-48%) compared with vehicle treatment. Cy/+ rats treated with 2DG also showed higher clearances of creatinine (1.98±0.67 vs 1.41±0.37 ml/min), BUN (0.69±0.26 vs 0.40±0.10 ml/min) and uric acid (0.38±0.20 vs 0.21±0.10 ml/min), and reduced albuminuria. Immunoblotting analysis of kidney tissues harvested from 2DG-treated Cy/+ rats showed increased phosphorylation of AMPK-α, a negative regulator of mTOR, and restoration of ERK signaling. Assessment of Ki-67 staining indicated that 2DG limits cyst progression through inhibition of epithelial cell proliferation. Taken together, our results show that targeting the glycolytic pathway may represent a promising therapeutic strategy to control cyst growth in PKD.

## Introduction

Autosomal dominant polycystic kidney disease (ADPKD) is the most common genetic renal disease, characterized by progressive development of innumerable cysts in both kidneys. The development and relentless growth of cysts lead to kidney enlargement and compression of renal parenchyma, leading to subsequent loss of renal function [1,2]. Recent studies have indicated that various signaling cascades are dysregulated in ADPKD, including activation of mTOR and downregulation of AMPK signaling, and enhanced vasopressin-mediated cAMP and ERK signaling [3,4,5,6]. Favourable results from several clinical trials targeting these pathways have led to the development of therapeutic concepts for ADPKD, yet treatment is not available for the majority of patients [7].

A promising strategy to achieve therapeutic selectivity and efficacy in ADPKD is to take advantage of the fundamental difference between cyst epithelial cells and normal epithelial cells in their energy metabolism. A recent study by Rowe et al. has shown that glycolysis was enhanced in a mouse model of ADPKD and also in kidney tissues from patients with ADPKD [8]. Metabolomic profiling of extracellular medium of embryonic fibroblast cultures derived from *Pkd1*^*-/-*^ and *Pkd1*^*+/+*^ mice indicated increased glucose uptake and lactate production and enhanced glycolytic enzyme gene expression in the mutant cells [8]. Using 2-deoxyglucose (2DG), an analog of glucose that blocks glycolysis, they found that cyst growth could be inhibited in rapidly progressing mouse models of polycystic kidney disease (PKD). Although the tested models did not allow for assessment of renal function, the data suggested that targeting glycolysis might represent a novel therapeutic strategy in ADPKD.

The purpose of the present study was to extend the above observations and to examine whether 2DG could be used in a chronic progressive rat model of PKD. Here we show that cystic kidneys in Han:SPRD rats display metabolic reprogramming in the sense of a ‘Warburg effect’ and that 2DG improved renal functional loss and cyst progression through normalization of intracellular signaling pathways.

## Methods

### Animals

The Han:SPRD rat colony was established in our animal facility from a litter which was obtained from the Rat Resource and Research Center (Columbia, MO, USA). Heterozygous cystic (Cy/+) and wild type normal (+/+) rats were used in this study. Han:SPRD rats carry a missense mutation in *Anks6* (also called *Pkdr1*), leading to an R823W substitution in SamCystin, a protein that contains ankyrin repeats and a sterile alpha motif (SAM) [9]. Han:SPRD Cy/+ rats develop a slowly progressing form of PKD which resembles phenotypically human ADPKD [10]. Only male rats were used in this study since cysts develop more rapidly in male compared with female rats [11]. The protocol has been approved by the committee on the Ethics of Animals Experiments at the University of Zurich (Licence Number: 174–2013). Rats had free access to tap water and standard rat diet.

### Genechip expression analysis

Affymetrix GeneChip^®^ rat genome 230 2.0 arrays were used according to the manufacturer's instructions (Affymetrix Inc., Santa Clara, CA, USA). Briefly, 5 μg of total RNA from 5-week old Cy/+ and +/+ rat kidneys was used as starting material to generate biotin-labeled cRNA samples, which includes cDNA synthesis using oligo-dT/T7 primers, followed by *in vitro* transcription (one-cycle labeling protocol). Labeled cRNA samples (15 μg) were randomly fragmented to 35–200 bp and hybridized on arrays for 16 h. After washing the arrays the fluorescent intensity emitted by the labeled targets was measured by an Affymetrix GeneChip^®^ Scanner 3000. Finally, the hybridization images were analyzed using Affymetrix GCOS 1.2 software.

### Reverse transcription and real-time PCR

RT-PCR analyses were performed as described previously [12,13]. Total RNA was reverse-transcribed and PCR was carried out using SYBR^®^ Green JumpStart Taq ReadyMix (Sigma-Aldrich, St Louis, MO, USA). Real-time PCR analyses were performed with the ABI PRISM 7500 Sequence Detection System (Applied Biosystems, Rotkreuz, Switzerland), according to the instructions of Applied Biosystems. The expression levels of β-actin were used as a housekeeping gene. Relative quantification of all targets was calculated by the comparative cycle threshold method outlined by the manufacturer (User Bulletin No. 2; Applied Biosystems, Rotkreuz, Switzerland).

### Experimental protocol

Male Cy/+ and +/+ rats were weaned and then treated at 5 weeks of age with 500 mg/kg/day 2DG (Cy/+: n = 10; +/+: n = 10) or vehicle NaCl (Cy/+: n = 10; +/+: n = 10) by daily subcutaneous injection for 5 weeks throughout the treatment phase. The dose of 2DG or vehicle was adjusted daily to the body weight of the rats. For blood collection, rats were anesthetized with inhalation of 1.5–3.5% isoflurane. Metabolic cages were used to collect 24-hour urine samples and to monitor food and fluid intake. Rats were acclimatized to the metabolic cage for an hour every day for three consecutive days prior to the actual metabolic cage experiment. All animals were sacrificed at week 10 by CO_2_ euthanasia.

### Blood and urine chemistries

Plasma and 24-hour urines were collected from rats at week 5, 7.5 and 10 and aliquots were rapidly frozen and stored at -80°C until measurement. Glucose, sodium, chloride, creatinine, blood urea nitrogen (BUN) and uric acid concentrations were determined in plasma and urine using a Cobas 8000 Modular Analyzer from Roche Diagnostics AG (Rotkreuz, Switzerland). Plasma and urine osmolality were measured by using an Advanced Osmometer Model 2020 (Advanced Instruments Inc., Norwood, MA, USA). Urinary albumin concentration was determined using a rat albumin ELISA kit (Genway, San Diego, CA, USA), as previously described [14]. Albuminuria was expressed as total urinary albumin excretion over 24-hour. Urine proteins were also analyzed by non-reducing SDS-PAGE and Coomassie blue staining.

### Tissue processing, periodic acid-Schiff staining, and cyst index measurement

At the age of 10 weeks, all rats were sacrificed and kidneys were excised, decapsulated and weighed. For histological examination, one of the kidneys from each animal was sliced perpendicularly to the long axis at approximately 2 mm intervals. Slices from the midportion of the kidneys were fixed in 4% buffered formalin and submitted to subsequent paraffin embedding. Serial sections of 3 μm thickness per paraffin block were cut and stained with periodic acid-Schiff (PAS) following a routine protocol. The stained sections were subjected to cyst index analysis, using the HistoQuest image analysis software (TissueGnostics, Vienna, Austria) to determine the cyst area (CA) and the total area (TA). The cyst index was calculated as CA/TA*100.

### Immunohistochemical detection of proliferation

Immunohistochemistry for Ki67 was performed on 3-μm tissue sections. In brief, the tissue sections were deparaffinized and rehydrated. The antigen retrieval was performed in an autoclave oven. Primary mouse anti-Ki67 antibody (BD Pharmingen, San Jose, CA, USA) and biotinylated secondary antibody (Vector, Los Angeles, CA, USA) were used. This was followed by the application of the ABC reagent, using 3,3’-diaminobenzidine with metal enhancement as the detection reagent. The stained sections were subjected to analysis using the HistoQuest image analysis software (TissueGnostics, Vienna, Austria) to quantify the number of Ki67-positive nuclei over the total area of the kidney section.

### Primary TEC cultures

Primary cultures of renal tubular epithelial cells (TECs) from 10-week-old +/+ and Cy/+ kidneys were prepared by mincing the kidneys and digesting the tissues with 1 mg/ml collagenase with gentle agitation for 1 h at 37°C. The suspension was allowed to sediment for 1 min. Cells were collected by harvesting the supernatant twice, and were then washed three times with 10% fetal bovine serum (FBS)/Hanks balanced salt solution. Isolated cells were resuspended in K1 medium (1:1 mixture of Dulbecco’s modified Eagle’s medium and Ham’s F-12 medium supplemented with 5% FBS, 10 mmol/l HEPES, 42 mmol/l sodium bicarbonate, 50 ng/ml insulin, 50 nmol/l hydrocortisone, 50 ng/ml transferrin, 5 pmol/l triiodothyronine, 20 ng/ml rat EGF, 100 IU/ml penicillin, and 100 μg/ml streptomycin). Cells were then seeded in collagen type 1–precoated culture dishes and grown to confluence. The medium was changed to a K1 medium with 0.5% FBS for 24 h, and the cells were then incubated with 2DG at various concentrations for 24 h. Cell viability was assessed by MTS assay following standard protocols.

Normal human kidney epithelial cells (NHK) were obtained from ATCC (ATCC^®^ PCS-400-010^™^, Manassas, VA, USA). ADPKD cells were prepared from nephrectomy material as previously described [15], after obtaining ethical approval from the ethics committee of the canton of Zurich, Switzerland, and informed oral consent. The culture conditions for ADPKD and NHK cells were similar to that of rat primary renal tubular epithelial cells.

### Western blotting analysis

Snap frozen kidney tissue was homogenized and tissue lysates or cell culture lysates were resolved by SDS–polyacrylamide gel electrophoresis, transferred to nitrocellulose membranes, and probed with primary antibodies. Mouse anti-Phospho-p70-S6K (Thr389), rabbit anti-p70-S6K mouse anti-Phospho-Erk1/2 (Thr202/Tyr24), rabbit anti-Erk1/2, rabbit anti-Phospho-AMPKα (Thr172), mouse-anti-Phospho-Akt (Ser473), rabbit anti-Akt and anti-caspase-3 antibodies were obtained from Cell signaling Technology, Beverly, MA, USA. Mouse anti-AMPKα was obtained from Santa Cruz Biotechnology (Santa Cruz, CA, USA). Anti-mouse and anti-rabbit secondary antibodies were purchased from Licor Biosciences (Lincoln, Nebraska, USA).

### ATP and lactate quantification

Intracellular ATP was quantified by luciferase activity according to the standard protocol in the ATP Determination kit (Invitrogen, Life Technologies, Zug, Switzerland). The concentration of lactate in cell culture supernatants and in kidney tissue from Han:SPRD rats was measured using the EnzyChrom L-lactate Assay Kit (BioAssay Systems, Hayward, USA), according to the manufacturer’s instructions.

### *In vitro* BrdU Proliferation Assay

*In vitro* proliferation of primary TEC was analyzed with the colorimetric cell proliferation BrdU assay kit (Roche Applied Science, Indianapolis, USA) according to the manufacturer’s instruction. Briefly, cells were cultured in 96-well plates with or without 2DG. After 48 hours, cells were labeled using 10 μM BrdU per well and re-incubated overnight at 37°C in a humidified atmosphere. The next day, the culture media was removed, the cells were fixed, and the DNA was denatured in one step by adding FixDenat solution. Cells were incubated with anti-BrdU-POD antibody, washed and the substrate solution was added. The reaction product was quantified by measuring the absorbance using a spectrophotometer at 370 nm with a reference wavelength of 492 nm.

### In vitro Cell Apoptosis Assay

Cells were cultured in 6-well plates with or without 2DG at a concentration of 1, 5, and 10 mM. After 24 hours, cells were collected following detachment with trypsin, resuspended in 140 mM NaCl, 10 mM HEPES, and 2.5 mM CaCl_2_ and incubated with annexin V-FITC (Roche Diagnostics, Basel, Switzerland) for 30 minutes at room temperature according to the manufacturer’s instructions. Flow cytometric analyses were performed using a BD-FACScan flow cytometer (BD Biosciences, San Jose, CA, USA). Data were analyzed using FlowJo software (Treestar Inc., Ashland, OR, USA.).

### Statistical analyses

All data are expressed as means ± SEM unless otherwise stated. Statistical analyses were performed using Student’s-*t-*test and ANOVA with Dunnett *post-hoc* test for multiple comparison analysis using GraphPad Prism version 5.0 (Graph Pad Inc., San Diego, CA, USA). A value of *P* <0.05 was considered as statistically significant.

## Results

### Increased glycolysis and decreased gluconeogenesis in polycystic kidney disease

**Fig 1:**
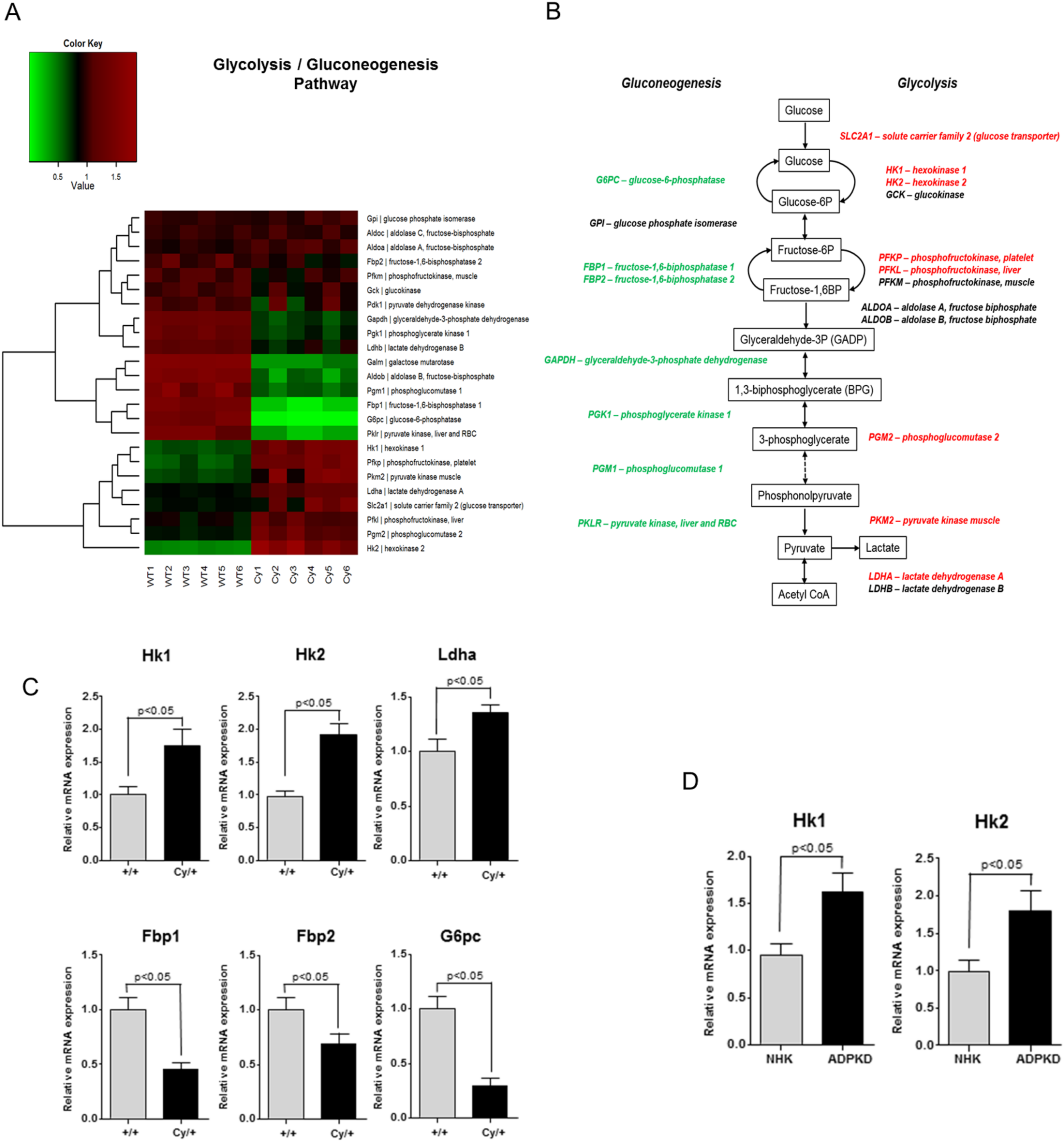
Dysregulation of the glycolysis and gluconeogenesis pathways in rat polycystic kidney disease. (A) Microarray analysis showing differential expression of genes coding for glycolysis and gluconeogenesis enzymes in Han:SPRD Cy/+ and wild-type +/+ kidneys. Upregulated genes are shown in red, and downregulated genes are shown in green. (B) Schematic diagram showing the glycolysis/gluconeogenesis cascades. In red, upregulated genes; green, downregulated genes; black, genes unchanged in kidneys from Cy/+ rats compared with wild-type +/+ kidneys. (C) Real-time quantitative PCR analysis of genes coding for key enzymes involved in glycolysis/gluconeogenesis in kidneys from Cy/+ rats and +/+ rats. (D) Real-time quantitative PCR analysis of the hexokinase-1 (Hk1) and hexokinase-2 (Hk2) genes in primary cell cultures of human ADPKD and control NHK cells. The expression levels of β-actin were used as a housekeeping gene.

First we performed microarray gene expression analysis on mRNA transcripts from kidney tissues from Han:SPRD Cy/+ and wild-type +/+ male rats to examine the glycolysis and the gluconeogenesis pathways of glucose metabolism. The microarray results are available online (accession number GSE75578) at the NCBI Gene Expression Omnibus (GEO). The mRNA levels of several genes involved in the glycolysis pathway were upregulated, whereas transcript levels of genes encoding for enzymes in gluconeogenesis were significantly donwregulated (Fig 1A and 1B). This observation was confirmed by quantitative real-time PCR analysis which demonstrated upregulation of key genes involved in the glycolysis pathway (*Hk1*, *Hk2*, *Ldha*) and downregulation of key genes involved in the gluconeogenesis pathway (*Fbp1*, *Fbp2*, *G6pc*) in the kidney tissues of Cy/+ rats versus +/+ rats (Fig 1C). Furthermore, upregulation of *Hk1* and *Hk2* genes was also observed in primary cell cultures of human ADPKD as compared to control primary cultures of normal human kidney (NHK) epithelial cells (Fig 1D).

**Fig 2:**
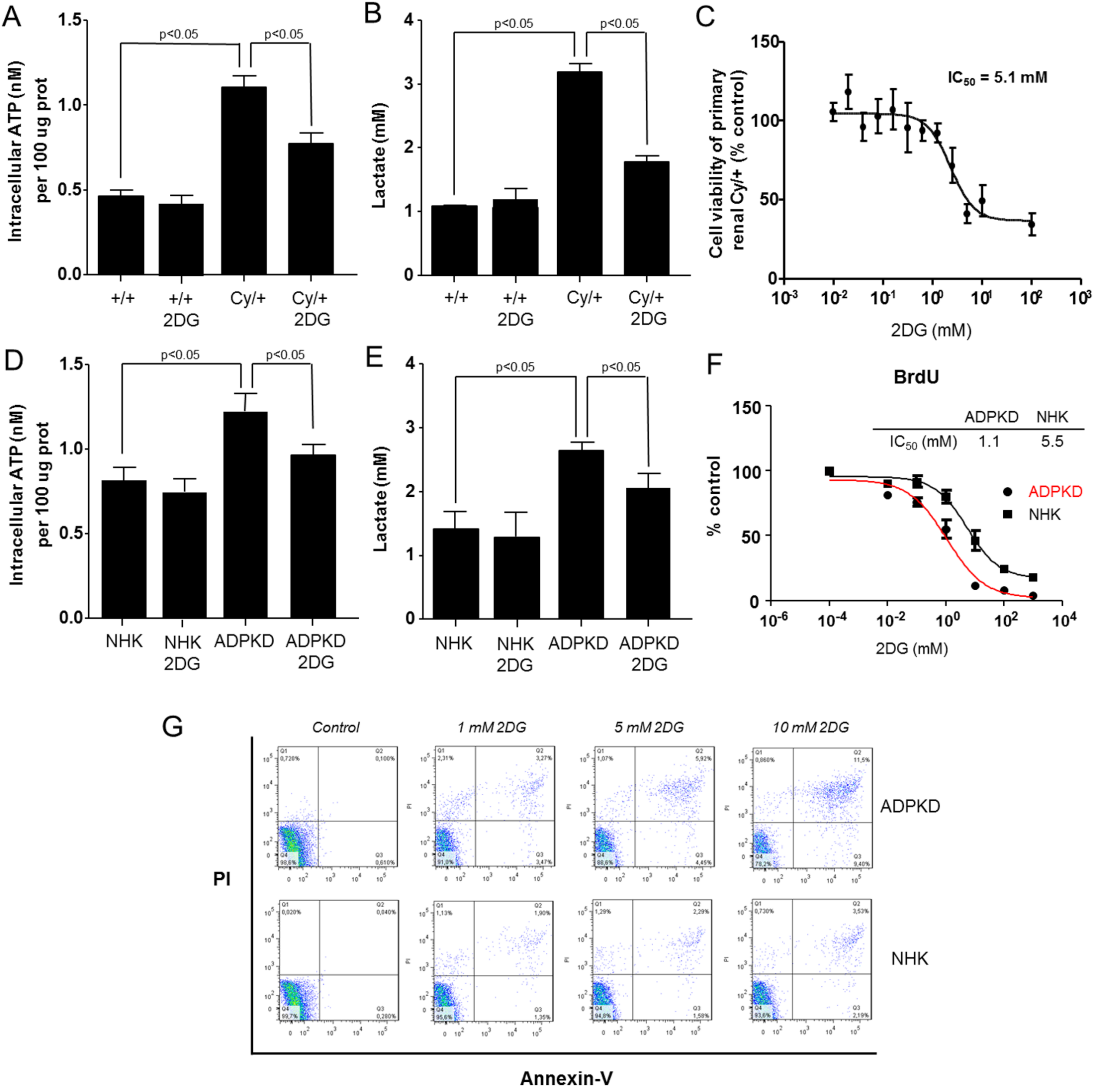
Increased glycolytic phenotype in polycystic kidney disease. (A) Intracellular ATP content in primary cell cultures of tubular epithelial cells isolated from kidneys of Cy/+ and +/+ rats. (B) Lactate concentration in the medium of primary cell cultures of tubular epithelial cells isolated from kidneys of Cy/+ and +/+ rats. (C) Cell growth of primary renal Cy/+ cells upon incubation with increasing concentrations of 2DG, as assessed by the MTS assay. (D) Intracellular ATP content in primary cell cultures of ADPKD and NHK cells. (E) Lactate concentration in the medium of primary cell cultures of ADPKD and NHK cells. (F) Effect of 2DG on cell proliferation of human ADPKD cells and control NHK cells, as quantified by BrdU assay. (G) Effect of 2DG on apoptosis of human ADPKD cells and control NHK cells, as analyzed with annexin-V/propidium iodide (PI) staining using flow cytometry.

To extend these observations we measured the intracellular ATP levels and the production of lactate *in vitro* using primary cell cultures of tubular epithelial cells isolated from kidneys of Cy/+ versus +/+ rats. The intracellular ATP levels were significantly increased in the Cy/+ cell cultures as compared to +/+ cell cultures, and were decreased upon incubation of Cy/+ cells with 2DG (Fig 2A). Furthermore, the lactate content in the cell culture media was higher in Cy/+ cell cultures in comparison to +/+ cell cultures, and was also decreased upon incubation of Cy/+ cells with 2DG (Fig 2B). No significant changes in the intracellular ATP levels or lactate content in the cell culture media were observed when +/+ cells were incubated with 2DG (Fig 2A and 2B). Similar observations were obtained in the primary cell cultures of human ADPKD and NHK cells; intracellular ATP levels and lactate in the cellular medium were higher in ADPKD cells as compared to NHK cells, and the levels were diminished upon incubation of ADPKD cells with 2DG (Fig 2D and 2E).

To analyze the effect of 2DG on cell proliferation and apoptosis we incubated primary renal tubular epithelial cells from Cy/+ rats with increasing concentrations of 2DG and found reduced cellular growth, as measured by the MTS assay (IC_50_ = 5.1 mM, Fig 2C). We also evaluated the effect of 2DG on cell proliferation and apoptosis of primary cultures of human ADPKD cells and control NHK cells. Incubation with 2DG limited cell proliferation (BrdU assay) and increased apoptosis (annexin-V staining) of primary human ADPKD cells in concentration-dependent manner (Fig 2F and 2G). Of note, the effects of 2DG on cell proliferation and apoptosis were more pronounced on ADPKD cells than on NHK cells, indicating a therapeutic potential of 2DG in human ADPKD (Fig 2F and 2G).

### *In vivo* effect of 2DG treatment

#### Effect of 2DG treatment on kidney weight and morphology

To assess the *in vivo* effect of glycolysis inhibition on cyst development and disease progression, we examined the effects of 2DG treatment in Han:SPRD Cy/+ and +/+ control rats. Rats were treated with 500 mg/kg/day 2DG or vehicle NaCl daily for 5 weeks. Based on the human equivalent dose [16], the amount of 2DG administered is equivalent to approximately 81 mg/kg/day in human. A previous study has reported 63 mg/kg/day to be the clinically tolerable dose for human clinical trials, with reversible side effects observed at a dose range of 63–88 mg/kg/day [17]. The dose used in the current proof-of-concept study should therefore not lead to any significant irreversible adverse effects.

**Fig 3:**
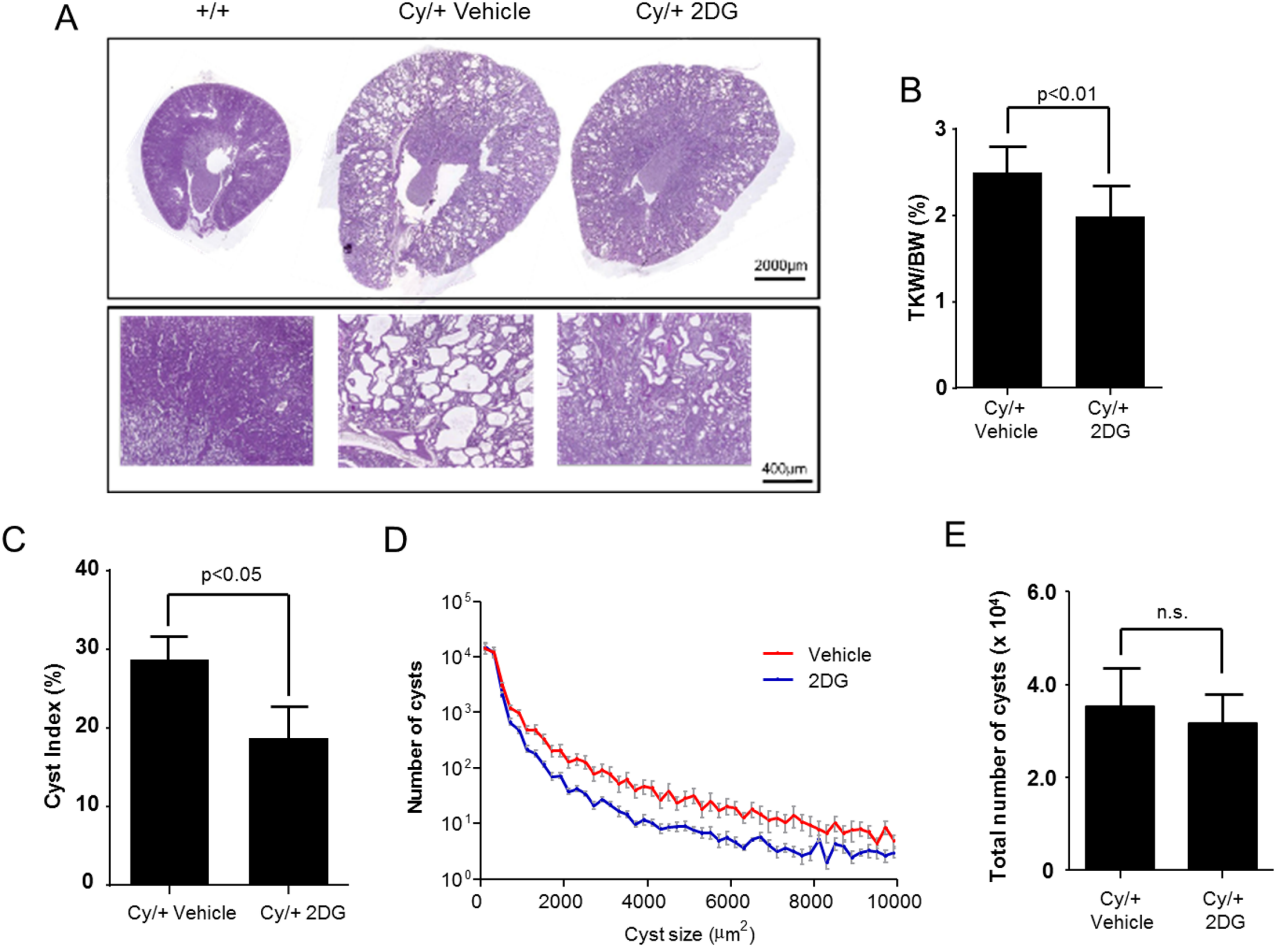
Effect of 2DG treatment on kidney weight and morphology in Han:SPRD Cy/+ rats. (A) Representative images of periodic acid-Schiff staining of kidneys from 10 week old +/+ and Cy/+ rats after 5-week treatment with 2DG or vehicle. (B) Ratio of total kidney weight (TKW) to body weight (BW) in 10 week old Cy/+ rats after 5-week treatment with 2DG or vehicle. (C). Cyst index in kidneys from Cy/+ rats after 5-week treatment with 2DG or vehicle. (D) Frequency distribution of the cyst size, and (E) total number of cysts, in kidneys from Cy/+ rats following 5-week treatment with 2DG or vehicle.

The 5-week treatment with 2DG was generally well tolerated in both animal groups. Side effects included lower body weight and mildly increased diuresis in 2DG-treated Cy/+ and +/+ rats. There were no significant changes in the plasma Na^+^ and Cl^−^, and no difference in plasma glucose levels between 2DG- and vehicle-treated Cy/+ and +/+ rats. At the end of the 5-week treatment phase, the total weight of both kidneys was significantly lower in 2DG-treated Cy/+ rats in comparison with vehicle-treated Cy/+ rats (-27.2%, *P*<0.001; Table 1). The 2-kidneys/body weight ratio was also lower upon 2DG treatment in Cy/+ rats as compared to vehicle treatment (-20.4%, *P*<0.01; Table 1, Fig 3A and 3B).

**Table 1:** Effect of 2DG treatment on kidney and body weight.

+/+						Cy/+				
	Baseline	Week 2.5		Week 5		Baseline	Week 2.5		Week 5	
		Vehicle	2DG	Vehicle	2DG		Vehicle	2DG	Vehicle	2DG
**Number of animals (n)**	20	10	10	10	10	20	10	10	10	10
**Age (in weeks)**	5	7.5	7.5	10	10		7.5	7.5	10	10
**Body Weight (g)**	161.4 (17.5)	284.7 (17.9)	266.8 (25.5)	339.2 (28.2)	328 (24.6)	167.4 (20.0)	290.1 (33.2)	263.1 (25.3)	345.0 (31.5)	317.1*(26.0)
**Total kidney weight (g)**				2.38 (2.66)	2.35 (1.75)				8.67 (1.46)	6.31***(1.14)
**%TKW/BW**				0.70 (0.06)	0.72 (0.04)				2.50 (0.29)	1.99**(0.30)

Data are presented as means and standard deviations (in parentheses).

*P<0.05,

**P<0.01,

***P<0.001 when comparing 2DG- versus vehicle-treated groups at each time point. TKW/BW, total kidney weight divided by body weight.

Histomorphological examination of the kidney sections showed that vehicle-treated Cy/+ rats had enlarged kidneys with multiple cysts which were not present in +/+ rats, as expected (Fig 3A). Treatment with 2DG resulted in a reduced size of the kidneys and reduced cyst sizes in Cy/+ rats compared with vehicle treatment (Fig 3A). Furthermore, quantification of the number of cysts on periodic acid Schiff-stained sections showed a significant reduction of the cyst index in 2DG-treated Cy/+ rats as compared to vehicle treatment (Fig 3C). Frequency distribution analysis of the cyst size showed that treatment with 2DG led to a shift in the size profile of the cysts (Fig 3D). The total number of cysts was similar in 2DG- and vehicle-treated rats (Fig 3E), suggesting that 2DG treatment affected cyst growth rather than cyst development in Cy/+ rats.

#### Effect of 2DG treatment on renal function

**Fig 4:**
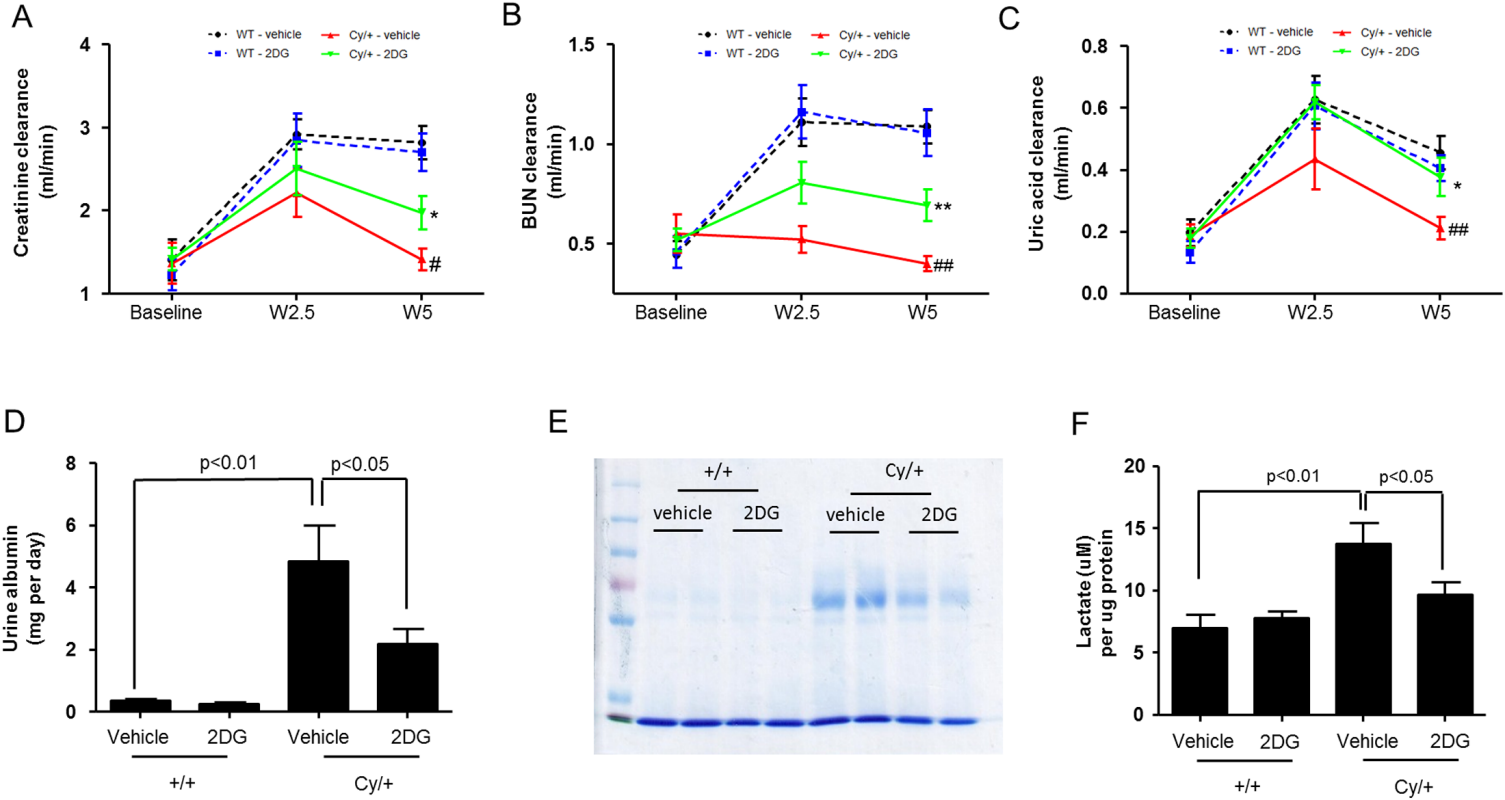
Effect of 2DG treatment on parameters of renal function. Measurement of (A) creatinine clearance, (B) BUN clearance, and (C) uric acid clearance, in +/+ and Cy/+ rats upon treatment with 2DG or vehicle at baseline, 2.5 weeks and 5 weeks. Black, +/+ treated with vehicle; blue, +/+ treated with 2DG; red, Cy/+ treated with vehicle; green, Cy/+ treated with 2DG. *P<0.05, **P<0.01 when comparing Cy/+ 2DG and Cy/+ vehicle at each time point. ^#^P<0.05, ^# #^ P<0.01 when comparing Cy/+ and +/+ group. (D) Urine albumin excretion in Cy/+ and +/+ rats after 5-week treatment with 2DG or vehicle. (E) SDS-polyacrylamide gel electrophoresis of urine samples from Cy/+ and +/+ rats after 5-week treatment with 2DG or vehicle. (F) Lactate content in the kidneys of Cy/+ and +/+ rats after treatment with 2DG or vehicle.

We then evaluated the effects of 2DG treatment on the parameters of renal function. Cy/+ rats developed significant impairment of renal function as compared to +/+ rats (Fig 4A, 4B and 4C). Following treatment with 2DG, Cy/+ rats had lower plasma creatinine levels as compared to vehicle treatment, with significantly higher clearances for creatinine (Fig 4A), BUN (Fig 4B) and uric acid (Fig 4C). Furthermore, the albumin excretion following treatment with 2DG was significantly lower as compared to vehicle-treated Cy/+ rats (Fig 4D and 4E). Lactate content in the kidneys of vehicle-treated Cy/+ rats were significantly higher than in vehicle-treated +/+ rats (Fig 4F). Treatment with 2DG reduced the level of lactate in the kidneys of Cy/+ rats when compared to vehicle treatment (Fig 4F).

#### Effect of 2DG treatment on cell proliferation and apoptosis

**Fig 5:**
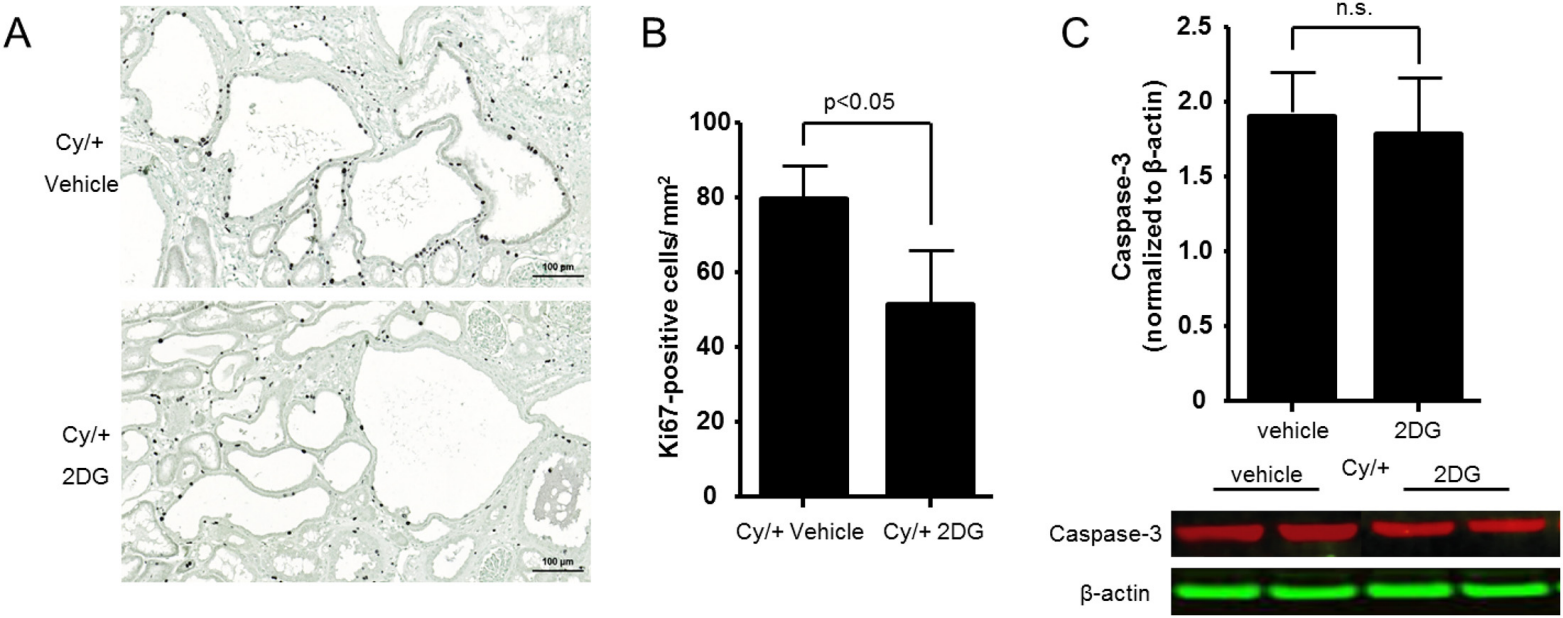
Effect of 2DG treatment on cell proliferation and apoptosis in Han:SPRD Cy/+ rats. (A) Representative images of Ki67 staining of cyst-lining epithelium in kidneys of Cy/+ rats treated with 2DG or vehicle. (B) Quantification of Ki67-positive nuclei in kidneys of Cy/+ rats after 5-week treatment with 2DG or vehicle. (C) Western blot analysis of active caspase-3 in kidneys from Cy/+ rats after treatment with 2DG or vehicle.

To examine whether 2DG reduces cyst epithelial cell proliferation we stained kidney sections for Ki67 and found a marked reduction in Ki67-positive nuclei in the tubular epithelium of Cy/+ kidneys as compared to vehicle treatment (Fig 5A and 5B). We also tested for apoptosis, examining active caspase-3 expression by Western blot analysis of kidney tissues from the Cy/+ rats. We found no difference in the level of active caspase-3 expression after treatment with 2DG as compared to vehicle treatment (Fig 5C). These data suggest that 2DG limits cyst growth in Cy/+ rats by inhibiting cell proliferation rather than by promoting apoptosis in the cystic epithelium.

#### Effect of 2DG treatment on cellular signaling pathways

**Fig 6:**
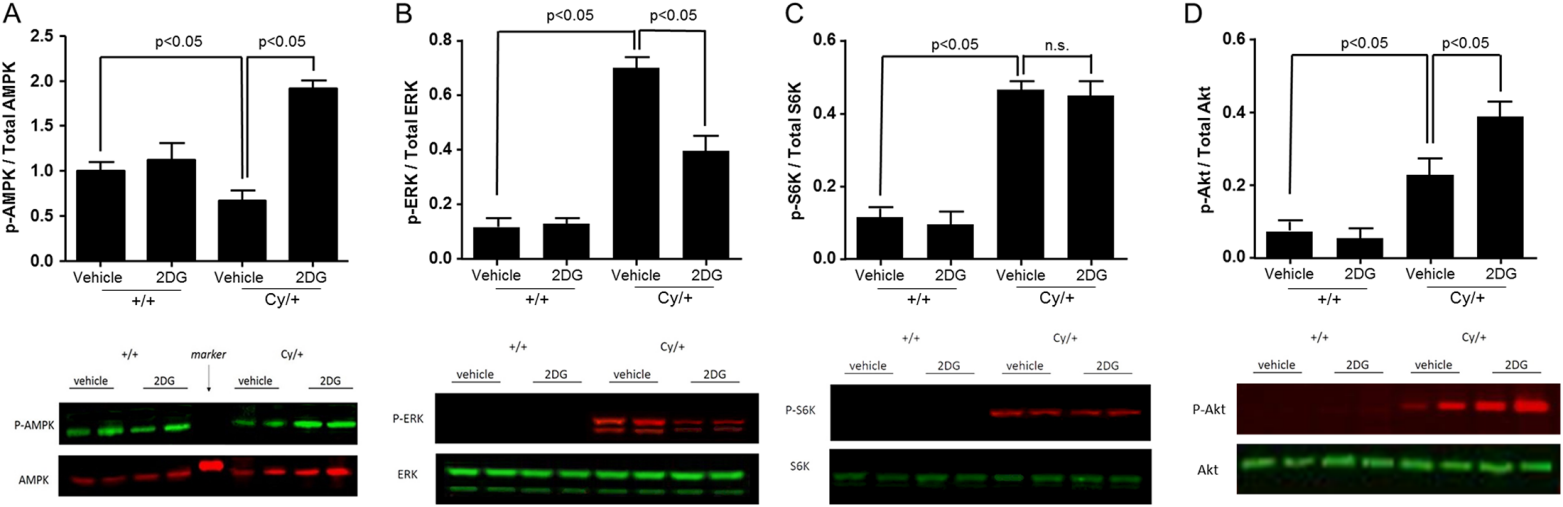
Effect of 2DG treatment on cellular signaling pathways *in vivo*. Measurements of phosphorylation levels of (A) AMPK, (B) ERK, (C) S6K, and (D) Akt using Western blot analysis in the kidneys of 10 week old +/+ and Cy/+ rats following 5-week treatment with 2DG or vehicle.

To further characterize the mechanisms of the increased glycolysis observed in Han:SPRD rats, we assessed the effect of 2DG on important signaling pathways that have previously been shown to be dysregulated in ADPKD. First, we examined the effect of 2DG on the phosphorylation of AMPK-α, an important negative regulator of mTOR, which has been shown to be reduced in PKD. Phosphorylation of AMPK-α was reduced in the kidney lysate from Cy/+ rats as compared to +/+ rats. Treatment with 2DG significantly increased AMPK-α phosphorylation in the kidneys of Cy/+ rats (Fig 6A). Next, we studied the effect of 2DG on the phosphorylation of two key kinases previously known to be activated in PKD, i.e. extracellular signal-regulated kinase (ERK; mitogen-activated protein kinase [MAPK] pathway) and p70 S6K (mTOR pathway). As shown in Fig 6B and 6C, phosphorylation of both ERK and p70 S6K were increased in the kidney of Cy/+ rats as compared to +/+ rats. Treatment with 2DG led to a significant reduction in the phosphorylation of ERK (Fig 6B), but there was no effect on the phosphorylation of p70 S6K (Fig 6C). Interestingly, 2DG treatment led to increased Akt phosphorylation (Fig 6D), which may likely explain the lack of an observable effect on p70 S6K as activation of Akt is known to counteract the effect of increased AMPK phosphorylation on p70 S6K activation.

**Fig 7:**
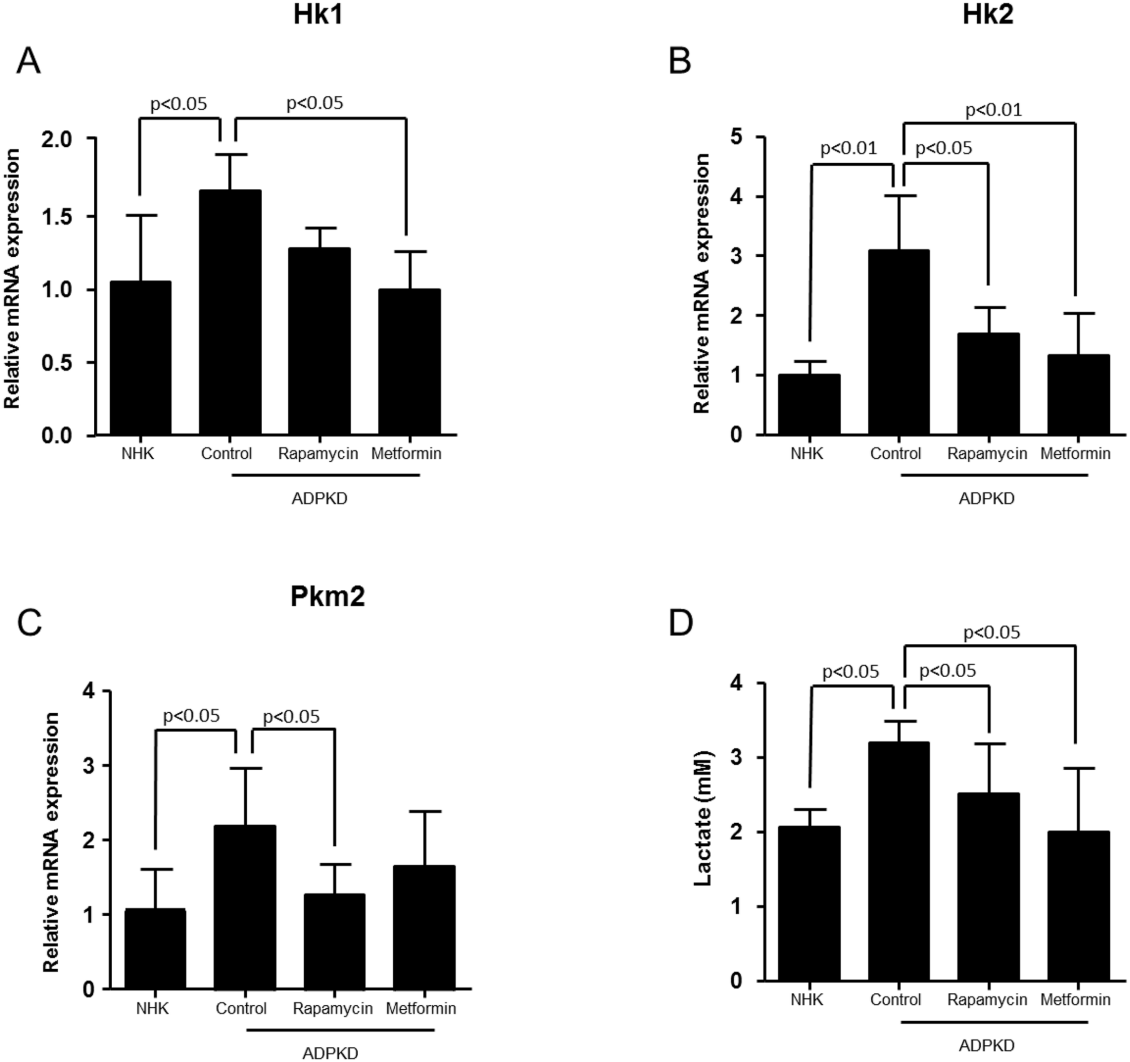
Effect of selected inhibitors on the glycolysis pathway *in vitro*. Real-time quantitative PCR analysis of the genes coding for glycolytic enzymes (A) Hk1, (B) Hk2, and (C) Pkm2 in primary cell cultures of human ADPKD cells following incubation with rapamycin (50 nM) or metformin (2 mM). (D) Lactate production in the supernatant of NHK and ADPKD cells, and response to inhibitors.

Previous studies have shown that activation of mTOR signaling is connected to enhanced glycolysis [18]. We therefore examined the effect of direct mTOR inhibition on the enhanced glycolysis which we observed in primary human cell cultures of ADPKD cells. Treatment with the mTOR inhibitor rapamycin (50 nM) reduced the enhanced mRNA expression of glycolytic genes (*Hk1*, *Hk2*, *Pkm2*) in ADPKD cells and also reduced the enhanced levels of lactate in the cell culture medium (Fig 7A–7D). In line with this, the expression of glycolytic genes and lactate production was also inhibited with the AMPK agonist metformin (2 mM), further emphasizing the importance of mTOR in glycolysis regulation (Fig 7A–7D).

## Discussion

In the present study we show that Han:SPRD Cy/+ rats display increased transcript levels for glycolysis enzymes and decreased transcript levels for gluconeogenesis enzymes in the kidney. Lactate and ATP levels were also increased in the kidneys and in TEC cultures of Cy/+ rats compared to wild type +/+ kidneys. Similar findings were obtained in cultured TEC derived from patients with ADPKD. Importantly, administration of 2DG, a glycolytic inhibitor, retarded cyst progression, improved renal function in Han:SPRD Cy/+ rats and was associated with reduced intrarenal lactate levels and normalization of altered signaling pathways. Taken together our data suggest that in PKD there is metabolic reprogramming towards enhanced aerobic glycolysis, a phenomenon which is known as the Warburg effect, after its discoverer Otto Warburg.

Recently, Rowe et al. reported that mutation of *Pkd1* results in enhanced glycolysis in a mouse model of PKD and in kidneys from patients with ADPKD [8,19]. Metabolomic profiling of the cell culture medium of *Pkd1*-deficient mouse embryonic fibroblasts showed increased glucose uptake and lactate production. The authors also found that treatment with 2DG delayed cyst growth in two rapidly progressive mouse models of PKD. However, the duration of the treatment with 2DG was extremely short (2 days), which does not account for the slowly progressive nature of the disease. In addition, the effect of 2DG on renal function could not be assessed due to the rapidly progressive nature of the models. More recently, Chiaravalli et al. used an orthologous and slowly progressive murine PKD model created by inducible inactivation of the Pkd1 gene postnatally and found that 2DG also had beneficial effects on cyst growth at a lower dose (100 mg/kg for 5 days per week for 2 months) [20].

Increased glycolysis has been previously reported in proliferating cells which require altered metabolism to efficiently incorporate nutrients such as glucose into biomass. Cancer cells, for instance, primarily metabolize glucose by glycolysis, whereas most normal cells completely catabolize glucose by oxidative phosphorylation [21]. This shift to aerobic glycolysis with increased lactate production, coupled with increased glucose uptake, is likely used by proliferating cells to promote the efficient conversion of glucose into the macromolecules needed to construct a new cell [22].

It has been shown that the Warburg effect is associated with defective mitochondrial respiration in the context of a hypoxic environment [23]. Similar to cancer cells, cyst epithelial cells in polycystic kidney disease are exposed to hypoxia and display mitochondrial dysfunction [24,25,26,27]. Furthermore it has been shown that cyst epithelial cells display a high rate of apoptosis, a process which is tightly regulated at the level of mitochondria [28]. Increased apoptosis in polycystic Han:SPRD rat kidneys was shown to be due to the activation of caspase-3 and dysregulation of the balance between pro- and anti-apoptotic Bcl-2 family members, specifically a down-regulation of anti-apoptotic Bcl-XL [29]. Zamzami et al. have shown that apoptosis is closely related to mitochondrial impairment [30]. There is also a high level of mitochondrial DNA (mtDNA) damage in apoptotic cells [31]. It is therefore conceivable that damage to the mtDNA would cause malfunction in the respiratory chain in PKD, forcing the cyst epithelial cells to use the aerobic glycolysis pathway to generate ATP.

The glucose analog 2-deoxyglucose (2DG) acts as a competitive inhibitor of glucose metabolism.[32] Upon transport into the cells, 2DG is phosphorylated by hexokinase to 2DG-P. However, unlike G-6-P, 2DG-P cannot be further metabolized by phosphohexose isomerase, which converts G-6-P to fructose-6-phosphate [33]. Thus 2DG-P is trapped and accumulates in the cells, leading to inhibition of glycolysis mainly at the step of phosphorylation of glucose by hexokinase. Inhibition of this rate-limiting step by 2DG causes a depletion of cellular ATP, leading to blockage of cell cycle progression and cell death *in vitro* [34]. The anti-tumor effect of 2DG has been well characterized in animal models and human clinical trials [35,36,37]. The feasibility of using a glycolytic inhibitor in a defined and progressive rat model of PKD has not been tested yet. In the current study, we investigated the therapeutic potential of 2-deoxyglucose (2DG) in Han:SPRD Cy/+ rats, a well-known animal model of PKD which develop slowly progressing renal cysts resembling human ADPKD.

A major complication of polycystic kidney disease is the deterioration of renal function. Glomerular filtration rate (GFR) significantly decreases towards the later stage of the disease, requiring the patients to initiate renal replacement therapy. Delaying the loss of renal function has therefore been a major therapeutic aim in PKD. In our study we show for the first time that 2DG improved different parameters of renal function (clearances for creatinine, BUN and uric acid). 2DG also ameliorated albuminuria, a common biomarker of altered and progressively deteriorating renal function in PKD. Taken together our data suggest that 2DG or related molecules may have a beneficial therapeutic potential for patients with ADPKD.

**Fig 8:**
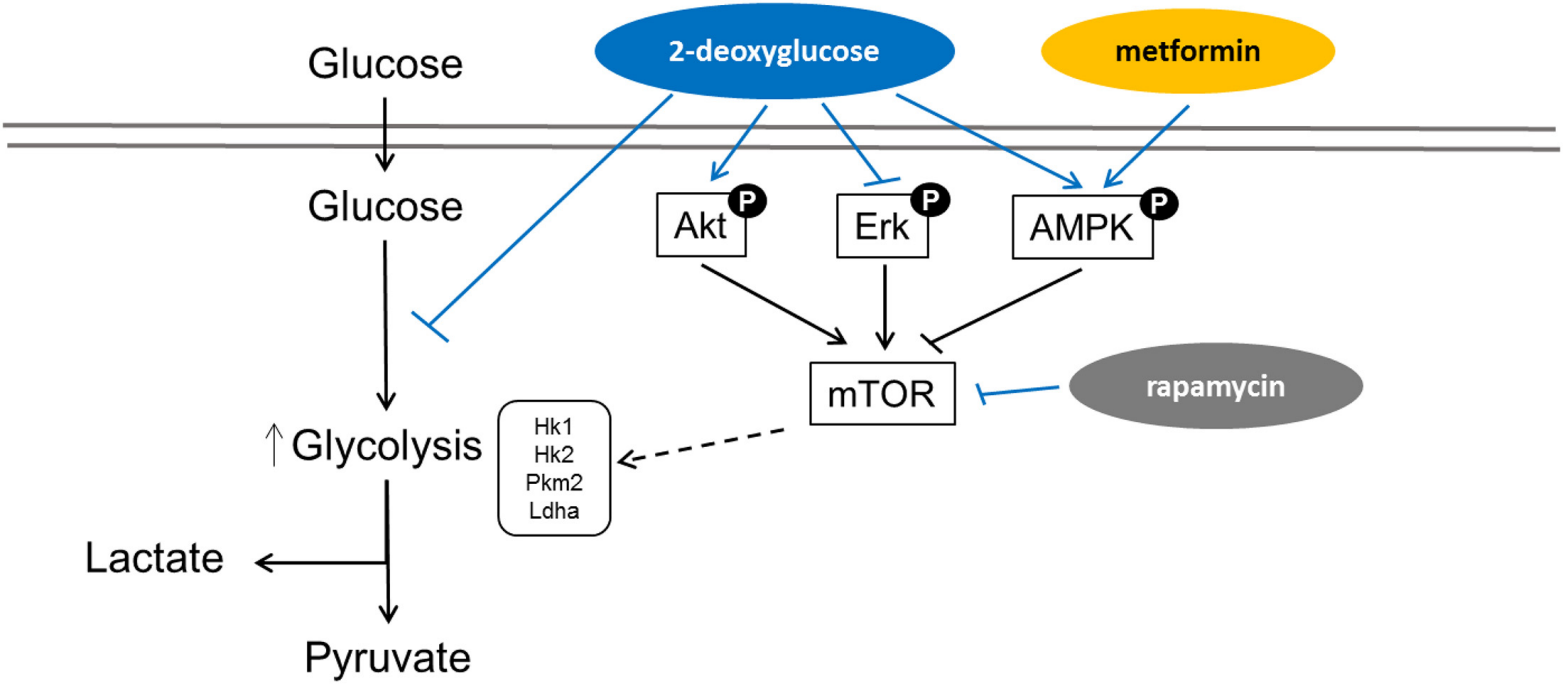
Schematic diagram showing the interplay of various signaling pathways involved in the regulation of glycolysis in polycystic kidney disease.

Interestingly, we observed that either direct inhibition of mTOR signaling using rapamycin or indirect inhibition using the AMPK agonist metformin reversed the glycolytic phenotype in ADPKD cells *in vitro*. Furthermore, we found that phosphorylation of ERK1/2 was significantly reduced in Cy/+ upon treatment with 2DG, which likely explains the observed effect of 2DG on cell proliferation. Interestingly, treatment of 2DG did not affect the phosphorylation status of S6K which is highly enhanced in Cy/+ kidneys as compared to wild-type rats. This could be explained by 2DG-mediated activation of the survival signal PI3K/Akt pathway, which counteracts the effect of 2DG via ERK and AMPK. As previously described, mTOR functions as a central node in a complex net of signaling pathways; it integrates signals from the Akt, ERK, and AMPK pathways and plays an important role in regulating protein biosynthesis, cellular growth, and proliferation [38,39]. Fig 8 summarizes the various signaling pathways involved in the regulation of glycolysis in ADPKD that have been investigated in this study.

Taken together, our results show that the cystic kidneys of Han:SPRD Cy/+ rats display enhanced aerobic glycolysis which likely plays an important role in the pathogenesis of PKD. The administration of 2DG, a potent glycolytic inhibitor, markedly delayed the loss of renal function and retarded cyst development Han:SPRD Cy/+ rats with PKD. Targeting the glycolytic pathway may therefore present a novel therapeutic strategy to control cyst growth in polycystic kidney disease.
